# Supplementary material for: Modeling glioblastoma heterogeneity as a dynamic network of cell states
Source: Mol Syst Biol. 2021 Sep 16;17(9):e10105. doi: 10.15252/msb.202010105 (PMC8444284; doi:10.15252/msb.202010105)
Supplement: Supplementary file 5 — Source Data for Figure 3 [file MSB-17-e10105-s001.zip › Figure3A_sourcedata/GSEA_3065/hallmarks_state6.GseaPreranked.1623416308702/HALLMARK_TNFA_SIGNALING_VIA_NFKB.html]

Details for gene set HALLMARK\_TNFA\_SIGNALING\_VIA\_NFKB[GSEA]

|  || Dataset | state6 |
| Phenotype | NoPhenotypeAvailable |
| Upregulated in class | na\_pos |
| GeneSet | HALLMARK\_TNFA\_SIGNALING\_VIA\_NFKB |
| Enrichment Score (ES) | 0.36018232 |
| Normalized Enrichment Score (NES) | 1.509894 |
| Nominal p-value | 0.016949153 |
| FDR q-value | 0.06754137 |
| FWER p-Value | 0.219 |
Table: GSEA Results Summary

  

Fig 1: Enrichment plot: HALLMARK\_TNFA\_SIGNALING\_VIA\_NFKB      
 Profile of the Running ES Score & Positions of GeneSet Members on the Rank Ordered List

  

| PROBE | GENE SYMBOL | GENE\_TITLE | RANK IN GENE LIST | RANK METRIC SCORE | RUNNING ES | CORE ENRICHMENT || 1 | CDKN1A |  |  | 1 | 0.477 | 0.0569 | Yes |
| 2 | GADD45A |  |  | 2 | 0.419 | 0.1071 | Yes |
| 3 | BTG1 |  |  | 4 | 0.348 | 0.1486 | Yes |
| 4 | PLK2 |  |  | 43 | 0.249 | 0.1739 | Yes |
| 5 | BTG2 |  |  | 63 | 0.234 | 0.1998 | Yes |
| 6 | PNRC1 |  |  | 123 | 0.202 | 0.2172 | Yes |
| 7 | KLF10 |  |  | 234 | 0.166 | 0.2244 | Yes |
| 8 | TSC22D1 |  |  | 398 | 0.136 | 0.2219 | Yes |
| 9 | EIF1 |  |  | 406 | 0.135 | 0.2373 | Yes |
| 10 | SNN |  |  | 473 | 0.126 | 0.2448 | Yes |
| 11 | ATF3 |  |  | 494 | 0.124 | 0.2573 | Yes |
| 12 | PHLDA1 |  |  | 612 | 0.113 | 0.2574 | Yes |
| 13 | BIRC2 |  |  | 644 | 0.111 | 0.2670 | Yes |
| 14 | LITAF |  |  | 692 | 0.106 | 0.2744 | Yes |
| 15 | SQSTM1 |  |  | 719 | 0.104 | 0.2838 | Yes |
| 16 | SAT1 |  |  | 744 | 0.102 | 0.2934 | Yes |
| 17 | NFE2L2 |  |  | 748 | 0.102 | 0.3052 | Yes |
| 18 | ETS2 |  |  | 793 | 0.099 | 0.3120 | Yes |
| 19 | NINJ1 |  |  | 819 | 0.097 | 0.3207 | Yes |
| 20 | DENND5A |  |  | 920 | 0.090 | 0.3200 | Yes |
| 21 | DUSP1 |  |  | 924 | 0.090 | 0.3304 | Yes |
| 22 | CEBPB |  |  | 943 | 0.089 | 0.3390 | Yes |
| 23 | PPP1R15A |  |  | 968 | 0.087 | 0.3466 | Yes |
| 24 | ID2 |  |  | 1058 | 0.082 | 0.3463 | Yes |
| 25 | MARCKS |  |  | 1081 | 0.081 | 0.3535 | Yes |
| 26 | CEBPD |  |  | 1166 | 0.077 | 0.3531 | Yes |
| 27 | SOD2 |  |  | 1228 | 0.075 | 0.3550 | Yes |
| 28 | CCND1 |  |  | 1317 | 0.071 | 0.3533 | Yes |
| 29 | KLF6 |  |  | 1424 | 0.067 | 0.3491 | Yes |
| 30 | GADD45B |  |  | 1497 | 0.064 | 0.3485 | Yes |
| 31 | ZBTB10 |  |  | 1502 | 0.064 | 0.3557 | Yes |
| 32 | TNIP1 |  |  | 1529 | 0.063 | 0.3602 | Yes |
| 33 | BHLHE40 |  |  | 1880 | 0.051 | 0.3261 | No |
| 34 | TNIP2 |  |  | 1911 | 0.050 | 0.3286 | No |
| 35 | RCAN1 |  |  | 1960 | 0.049 | 0.3289 | No |
| 36 | PDE4B |  |  | 2004 | 0.048 | 0.3297 | No |
| 37 | BTG3 |  |  | 2012 | 0.047 | 0.3346 | No |
| 38 | MYC |  |  | 2081 | 0.045 | 0.3322 | No |
| 39 | SLC2A3 |  |  | 2103 | 0.045 | 0.3351 | No |
| 40 | FOSL2 |  |  | 2129 | 0.044 | 0.3375 | No |
| 41 | KDM6B |  |  | 2138 | 0.044 | 0.3418 | No |
| 42 | IRS2 |  |  | 2158 | 0.043 | 0.3447 | No |
| 43 | JAG1 |  |  | 2234 | 0.040 | 0.3409 | No |
| 44 | NFAT5 |  |  | 2267 | 0.039 | 0.3420 | No |
| 45 | BCL6 |  |  | 2351 | 0.037 | 0.3369 | No |
| 46 | IRF1 |  |  | 2377 | 0.037 | 0.3384 | No |
| 47 | PMEPA1 |  |  | 2388 | 0.036 | 0.3416 | No |
| 48 | EHD1 |  |  | 2661 | 0.030 | 0.3139 | No |
| 49 | PLPP3 |  |  | 2894 | 0.024 | 0.2901 | No |
| 50 | RELA |  |  | 2971 | 0.023 | 0.2840 | No |
| 51 | TRIP10 |  |  | 2989 | 0.022 | 0.2847 | No |
| 52 | FOSL1 |  |  | 3108 | 0.020 | 0.2735 | No |
| 53 | SDC4 |  |  | 3129 | 0.019 | 0.2735 | No |
| 54 | ABCA1 |  |  | 3161 | 0.019 | 0.2722 | No |
| 55 | MCL1 |  |  | 3430 | 0.012 | 0.2428 | No |
| 56 | CFLAR |  |  | 3461 | 0.012 | 0.2408 | No |
| 57 | TGIF1 |  |  | 3672 | 0.007 | 0.2175 | No |
| 58 | SERPINE1 |  |  | 3811 | 0.004 | 0.2022 | No |
| 59 | PLAUR |  |  | 3956 | 0.001 | 0.1858 | No |
| 60 | SOCS3 |  |  | 3978 | 0.001 | 0.1835 | No |
| 61 | JUNB |  |  | 4044 | -0.000 | 0.1760 | No |
| 62 | SMAD3 |  |  | 4246 | -0.004 | 0.1534 | No |
| 63 | CSF1 |  |  | 4350 | -0.006 | 0.1423 | No |
| 64 | REL |  |  | 4354 | -0.006 | 0.1426 | No |
| 65 | YRDC |  |  | 4409 | -0.007 | 0.1373 | No |
| 66 | FOS |  |  | 4536 | -0.010 | 0.1239 | No |
| 67 | TANK |  |  | 4755 | -0.014 | 0.1005 | No |
| 68 | NFKB1 |  |  | 4949 | -0.017 | 0.0803 | No |
| 69 | G0S2 |  |  | 4967 | -0.018 | 0.0805 | No |
| 70 | DUSP4 |  |  | 5000 | -0.018 | 0.0790 | No |
| 71 | DRAM1 |  |  | 5008 | -0.018 | 0.0804 | No |
| 72 | PLAU |  |  | 5312 | -0.025 | 0.0485 | No |
| 73 | TIPARP |  |  | 5417 | -0.027 | 0.0397 | No |
| 74 | VEGFA |  |  | 5660 | -0.032 | 0.0157 | No |
| 75 | PTPRE |  |  | 5684 | -0.032 | 0.0169 | No |
| 76 | IER2 |  |  | 5689 | -0.032 | 0.0203 | No |
| 77 | B4GALT5 |  |  | 5833 | -0.035 | 0.0080 | No |
| 78 | B4GALT1 |  |  | 5870 | -0.036 | 0.0081 | No |
| 79 | RIPK2 |  |  | 6203 | -0.043 | -0.0250 | No |
| 80 | SLC2A6 |  |  | 6321 | -0.045 | -0.0330 | No |
| 81 | EGR1 |  |  | 6519 | -0.050 | -0.0497 | No |
| 82 | PDLIM5 |  |  | 6585 | -0.052 | -0.0509 | No |
| 83 | MAP2K3 |  |  | 6986 | -0.064 | -0.0892 | No |
| 84 | IFNGR2 |  |  | 6997 | -0.065 | -0.0826 | No |
| 85 | NFKBIA |  |  | 7027 | -0.066 | -0.0781 | No |
| 86 | TUBB2A |  |  | 7078 | -0.067 | -0.0758 | No |
| 87 | CCNL1 |  |  | 7107 | -0.068 | -0.0709 | No |
| 88 | NAMPT |  |  | 7188 | -0.071 | -0.0716 | No |
| 89 | BMP2 |  |  | 7299 | -0.074 | -0.0754 | No |
| 90 | HES1 |  |  | 7301 | -0.074 | -0.0666 | No |
| 91 | RHOB |  |  | 7306 | -0.075 | -0.0581 | No |
| 92 | IER5 |  |  | 7383 | -0.077 | -0.0576 | No |
| 93 | JUN |  |  | 7513 | -0.082 | -0.0627 | No |
| 94 | LDLR |  |  | 7648 | -0.088 | -0.0675 | No |
| 95 | F2RL1 |  |  | 7728 | -0.093 | -0.0655 | No |
| 96 | FJX1 |  |  | 8110 | -0.115 | -0.0957 | No |
| 97 | PTX3 |  |  | 8319 | -0.134 | -0.1035 | No |
| 98 | TAP1 |  |  | 8393 | -0.146 | -0.0945 | No |
| 99 | IL6ST |  |  | 8394 | -0.146 | -0.0770 | No |
| 100 | TNFAIP6 |  |  | 8434 | -0.150 | -0.0635 | No |
| 101 | ATP2B1 |  |  | 8436 | -0.150 | -0.0457 | No |
| 102 | CD44 |  |  | 8778 | -0.337 | -0.0446 | No |
| 103 | TNC |  |  | 8787 | -0.389 | 0.0010 | No |
Table: GSEA details [plain text format]

  

Fig 2: HALLMARK\_TNFA\_SIGNALING\_VIA\_NFKB: Random ES distribution      
 Gene set null distribution of ES for **HALLMARK\_TNFA\_SIGNALING\_VIA\_NFKB**
